# High-throughput method for Antibiotic Susceptibility Testing based on Fluorescein Quenching by Bacteria: Application to Urinary Tract Infection

**DOI:** 10.1038/s41598-020-60717-9

**Published:** 2020-03-04

**Authors:** Rohit Radhakrishnan, Rajesh J., Dinesh N. S., Thangavelu C. P., Sankaran K.

**Affiliations:** 1 Centre for Biotechnology, Anna University, Chennai, 600 025 India; 2 Department of Electronic Systems Engineering, Indian Institute of Science, Bangalore, 560 012 India; 3 Microbiological Laboratory, Coimbatore, Tamil Nadu 641 002 India

**Keywords:** Bacterial infection, Diagnosis

## Abstract

We recently reported a sugar-induced bacterial release of 13-Docosenamide and its ability to quench fluorescein. This simple handle to monitor bacterial growth is readily applicable to develop a quicker antibiotic sensitivity testing method along with a low-cost field-use optical instrumentation. Conditions were standardized to perform this new procedure in the most preferred and CLSI-recommended microdilution format in 12-well strips. A simple and portable optoelectronic prototype was used to capture the image and read the fluorescence signal of the culture medium of the 12-well strips. This new Fluorescence Quenching Method along with the device enabled the choice of the right antibiotic within 8 h of sample collection from the patient. It was compliant to the Clinical Laboratory Standard Institute’s quality control guidelines. Clinical assessment of the method using 440 urine samples from Urinary Tract Infection patients against 21 routinely used antibiotics showed a 94.3% match with the results of the Standard Disk Diffusion method. This new method saves the precious time taken for and the cost of antibiotic susceptibility testing for quicker and effective treatment with better compliance.

## Introduction

Among the many factors attributed to the rise of antibiotic resistance, prescription of antibiotics without susceptibility testing (AST) is the primary and the most alarming one^1^. In countries like India, and even in developed countries, this is a common practice, except that in the latter case, antibiotic prescriptions are restricted and required for the purchase. Lack of simple and cost-effective AST performed even in peripheral clinics to obtain results within a few hours is the sole reason for the continuation of this risky practice.

The current diagnostic tests available to determine the antibiotic sensitivity do not meet the needs of the developing countries and several pockets in the developed countries. The conventional agar-based Disk diffusion method and E-Test of AST determination can benefit from newer developments^2^. The current automated systems require infrastructure and skill sets that are unattainable by many laboratories. The manual method requires augmented culture techniques which are time-consuming typically returning a susceptibility profile in 48 h or longer^3^. The broth microdilution method, a Clinical and Laboratory Standards Institute (CLSI) reference method, which is the “gold standard” AST method, requires incubation of 16–24 hours^4^. After incubation, the results are read visually or by measuring OD_600 nm_. Even with automated system and technologies which reduce the working time to about 50%^5^, the current methods do not provide the relevant information in time before the initiation of the first antibiotic therapy or even by the second dose.

We had recently published that *Escherichia coli* and 26 other bacteria associated with human diseases when grown in glucose-containing medium, released long-chain fatty amide(s) of the type 13-docosenamide, which quenched fluorescein, a widely-used inexpensive fluorophore. This quenching of the fluorescence of fluorescein was directly proportional to the growth of bacterial culture^6^. Thus, an absence in the fluorescence of the growth medium indicates bacterial growth while the fluorescence of the uncultured medium remains intact. We adapted this bacterial growth monitoring technique that based on the optical shift, for developing a simpler and quicker AST (within 8 h) as reported here. This Fluorescence Quenching Method (FQM) is performed in micro-wells of commercially available 12-well strips or plates. The fluorescence of the medium is captured using an optical imager fabricated in-house for 12-well strips.

## Results

### Fluorescence Quenching Method (FQM) results matched CLSI quality control range for four reference ATCC bacterial strains

The compliance of FQM to CLSI guidelines was tested with four CLSI recommended reference ATCC bacterial strains against a panel of 10 different antibiotics, prepared according to the MIC ranges defined by CLSI (Table 1). The MIC results determined using FQM (Tryptic Soy Broth containing Glucose and Fluorescein, TSB-GF medium) for the ATCC strains were compared with that of the CLSI Broth Microdilution method (MH broth). The MIC of each antibiotic for the four ATCC strains determined by FQM and CLSI microdilution method was the same and within CLSI defined quality control range (Table 1). This absolute match was repeatable in 15 consecutive trials, each performed once daily. The fluorescence value of the medium, TSB-GF, and that of the susceptible samples did not alter significantly from 8 h to 24 h of testing indicating excellent stability of the dye in the medium even for extended incubations. The culture without antibiotic and at concentrations below MIC, the fluorescence decreased in proportion to the growth at 8 h, the cut off time for FQM, and beyond.

**Table 1:** MIC (mg/L) for ATCC bacterial strains determined using CLSI microdilution method and FQM against antibiotics prepared with different concentrations according to CLSI quality control ranges.

S.no	Antibiotics	Method	*E. coli* (25922)		*E. faecalis* (29212)		*P. aeruginosa* (27853)		*S. aureus* (29213)	
			CLSI QC Range	MIC	CLSI QC Range	MIC	CLSI QC Range	MIC	CLSI QC Range	MIC
1	**Amikacin (AK)**	**CLSI**	4.0-0.5	1.0	256-64	64	4.0-1.0	2.0	4.0-1.0	2.0
		**FQM**		1.0		64		2.0		2.0
2	**Ampicillin (AMP)**	**CLSI**	8.0-2.0	4.0	2.0-0.5	0.5	Cannot Be Used		2.0-0.5	0.5
		**FQM**		4.0		0.5				0.5
3	**Cefotaxime (CTX)**	**CLSI**	0.12-0.03	0.06	Cannot Be Used		32-8.0	8.0	4.0-1.0	1.0
		**FQM**		0.06				8.0		1.0
4	**Ceftriaxone (CTR)**	**CLSI**	0.12-0.03	0.06	Cannot Be Used		64-8.0	32	8.0-1.0	1.0
		**FQM**		0.06				32		1.0
5	**Ciprofloxacin (CIP)**	**CLSI**	0.016-0.004	0.016	2.0-0.25	0.25	1.0-0.25	0.25	0.48-0.12	0.12
		**FQM**		0.016		0.25		0.25		0.12
6	**Gentamicin (GEN)**	**CLSI**	16-4.0	0.050	16-4.0	16	2.0-0.5	1.0	1.0-0.12	1.0
		**FQM**		0.050		16		1.0		1.0
7	**Norfloxacin (NX)**	**CLSI**	0.12-0.03	0.06	8.0-2.0	2.0	4.0-1.0	1.0	2.0-0.5	1.0
		**FQM**		0.06		2.0		1.0		1.0
8	**Ofloxacin (OF)**	**CLSI**	0.12-0.015	0.06	4.0-1.0	1.0	8.0-1.0	1.0	1.0-0.12	0.12
		**FQM**		0.06		1.0		1.0		0.12
9	**Piperacillin (PI)**	**CLSI**	4.0-1.0	2.0	4.0-1.0	1.0	8.0-1.0	4.0	4.0-1.0	1.0
		**FQM**		2.0		1.0		4.0		1.0
10	**Nitrofuran-toin (NIT)**	**CLSI**	16-4.0	4.0	16-4.0	4.0	Cannot Be Used		32-8.0	8.0
		**FQM**		4.0		4.0				8.0

### A simple and inexpensive fluorescence imaging device enabled field-use of FQM

**Figure 1:**
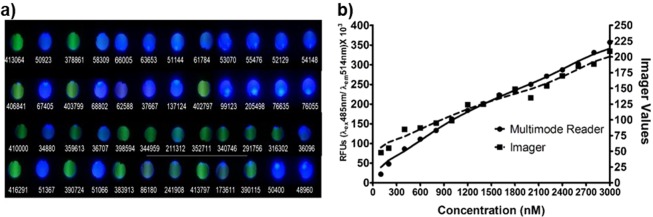
(**a**) Images of isolates cultured by FQM captured using the imager after a static incubation period of 8 h. The corresponding multimode reader values are indicated below each well. (**b**) The imager values for different concentrations of fluorescein compared with the corresponding fluorescence response (RFU) of a commercial fluorescence reader.

The low-cost portable imager (see materials and methods) prototype generated the image of the fluorescence pattern in a 12-well test strip as given in Fig. 1a. The medium control wells appeared green owing to the intense fluorescence of fluorescein. The wells with growth were blue to bluish-green depending upon the amount of the quencher produced in proportion to the growth and the image processing software calculates the amount of green colour in each well and represents it with a number ranging from 0–256.

The image processing software was calibrated with standard fluorescein concentration range 0 nM–3000 nM. The values generated by the software for the different concentration of fluorescein was compared with the fluorescence reading (RFUs) values of the commercial multi-mode reader. The setting at which the linear response of the green values determined by the algorithm matched that of the commercial reader values was fixed for measuring the signal (Fig. 1b). Hence, both the imager values and the reader values were proportional to the accumulative intensity of increasing concentration of fluorescein in the solution. This setting had been verified thoroughly by routine and several repeatability tests.

### FQM proved to be clinically useful AST procedure for urine samples

FQM, along with the imaging device, was evaluated for its performance as an AST tool at the Microbiological Laboratory, Coimbatore, Tamil Nadu, India, which is accredited by National Accreditation Board for Testing and Calibration Laboratories.

Among 440 urine samples tested, 20 were sterile and six contained *Candida* spp. Both did not quench the fluorescence of TSB-GF medium. Thirty-three samples with a colony count below 1 × 10^3^ CFU/mL (taken as borderline and clinically insignificant) was not detected by FQM within 8 h incubation period. However, the growth and quenching could be detected conveniently on longer and overnight incubation.

Apart from the sterile samples, 381 urine samples with significant bacteriuria were tested against 21 antibiotics by FQM at three concentrations. This provided 8001 results on antibiotic susceptibility. The same samples tested by Standard Disk Diffusion (SDD) against 13 antibiotics (in discs) provided 5027 (Table 2) results for comparison with the popular clinical method. Among these, 3856 points (76.7%) in agreement, 860 (17.1%) showed a shift in MIC values categorized as inconsequential minor error (mismatch in one concentration within the range). Hence, practically, the match between the two methods was 93.8%. 311 points (6.2%) showed major and very major errors between the methods; The major errors were 165 points (3.2%) majority (1.4%) of which is due to Colistin (match was confirmed with CLSI microdilution); very major errors were 146 points (3.0%) mainly contributed by Meropenem (1.0%).

**Table 2:** Comparison of antibiotic susceptibility results for clinical samples determined using FQM and SDD method.

S/N	Antibiotics	Total no: of comparisons*	Match (%)	Minor Error (%)	Major Error (%)	Very Major Error (%)
1	**Amikacin (AMI)**	354	67.89	27.95	4.16	—
2	**Amoxycillin/clavulanate (AMC)**	349	89.04	7.08	2.45	
3	**Ampicillin (AMP)**	57	100	—	—	—
4	**Ceftazidime (TAZ)**	8	100	—	—	—
5	**Cefepime (FEP)**	345	78.55	11.72	6.26	3.47
6	**Cefixime (CEF)**	327	75.78	8.50	11.85	3.88
7	**Ceftriaxone (AXO)**	349	79.87	15.08	3.62	1.43
8	**Ciprofloxacin (CIP)**	354	80.80	6.13	7.69	5.38
9	**Colistin (COL)**	343	73.94	6.12	19.94	—
10	**Erythromycin (ERY)**	4	100	—	—	—
11	**Gentamicin (GEN)**	308	79.61	16.41	2.51	1.47
12	**High Level Gentamicin (HGN)**	49	92.56	2.44	2.32	2.68
13	**Imipenem (IMI)**	359	91.57	1.89	6.54	—
14	**Levofloxacin (LEV)**	5	100	—	—	—
15	**Meropenem (MER)**	354	77.29	5.88	2.86	13.97
16	**Nitrofurantoin (NIT)**	372	63.77	27.10	5.40	3.73
17	**Norfloxacin (NOR)**	292	82.74	7.02	9.14	1.10
18	**Piperacillin/Tazobactam (TZP)**	369	82.24	10.45	2.21	5.10
19	**Tetracycline (TET)**	48	94.24	5.76	—	—
20	**Tigecycline (TGC)**	376	95	—	5.00	—
21	**Vancomycin (VAN)**	5	100	—	—	—
	**Total test points**	5027	76.7% (3856)	17.1% (860)	3.2% (165)	3.0% (146)

(*Number of comparisons for each antibiotic varies because SDD method uses different panels of antibiotics based on the identity of pathogens, whereas FQM blindly tested for all 21 drugs listed in the table).

These results show FQM employing direct clinical samples of urine could give reliable antibiogram results after 8 h aerobic incubation when there is a single pathogen causing urinary tract infection, which is the case in the majority of samples. Among the infected samples, *E. coli* was the most prevalent, accounting for 75% of the 381 samples and 8 other bacteria together constitute the rest 25% (Table 3).

**Table 3:** Prevalence ratio of different bacterial species among the clinical samples tested for AST in this study.

S/N	Bacterial Species	No. of organism tested	Prevalence (%)
1	*Escherichia coli*	285	74.9
2	*Klebsiella pneumoniae*	25	6.6
3	*Enterococcus* spp.	25	6.6
4	*Citrobacter diversus*	18	4.7
5	*Klebsiella oxytoca*	9	2.3
6	Non-Fermenting Gram negative bacilli	9	2.3
7	*Pseudomonas aeruginosa*	5	1.3
8	*Proteus mirabilis*	4	1.0
9	*Serratia spp*.	1	0.3
**Total No. of tests performed**		381	

## Discussion

Increasing prevalence of antibiotic resistance among community infections, time-consuming and costlier microbiological investigations demand quicker and affordable ways of knowing the right antibiotic for treatment. Survey among the Indian physicians of peripheral hospitals and private clinics catering to the upper and lower middle-class populations indicated the need for the right choice of antibiotic first within 6–8 h, even before pathogen identification, especially targeting urinary tract infections (UTI). The test results of the clinical samples reported here showing ~30% of them being resistant to 3–4 and ~12% to 9–10 types of commonly prescribed antibiotics justify such a demand. Based on the survey input, we preferred to perform AST for UTI either prior to or simultaneously with the time-consuming procedure of isolation and identification of the pathogens. Our recent discovery of growth-dependent bacterial secretion 13-Docosanamide which quenches the fluorescence of fluorescein^6^ offered a simple AST approach with associated inexpensive fluorescence imaging instrumentation.

After extensive standardization and optimizations to suit the field conditions, we found that the bacterial growth in the ready-to-use fluorescent medium (TSB-GF) was accompanied by significant quenching by 4 h and reached 70–90% by 8 h of incubation for a variety of clinical bacteria. Our low-cost optoelectronic-imaging device equipped with our own image processing software was comparable to a standard commercial microplate fluorescence reader in terms of quantitation and accuracy of AST results. Since fluorescein is a very stable fluorophore, quenching above 20% of its initial fluorescence was taken as bacterial growth. Hence, the wide fluorescence quenching range (from 20% to 100%) available during 8 h growth period, accommodated errors and variations typical of biological measurements and assays in reporting bacterial growth. The laboratory-level and the clinical evaluation described here have clearly borne out these advantages. The TSB-based medium prepared with fluorescein, highly fluorescent and inexpensive fluorophore was found to be stable for months^7–9^ even at room temperature when stored in dark.

In the evaluation of FQM with clinical urine samples, the discordance of 6.2% (interpreted with CLSI breakpoints) with disk diffusion was akin to those reported for the established liquid-based antibiotic susceptibility assays. Particularly, Colistin^10,11^ and Meropenem^12,13^ known for the anomalous behaviour in disk diffusion. For Colistin, CLSI broth microdilution method is recommended due to its diffusibility problem from the disks^14^. For meropenem, it could be due to the high instability of this β-lactam derivative in aqueous solutions as well as in solid-state^15^ even under the manufacturer’s recommended storage conditions. This explains that the discordance is towards susceptibility for FQM in which fresh antibiotic stocks were used versus resistance in SDD. We had also verified our results with the CLSI broth microdilution method and plating on Meropenam agar plates. The CLSI recommended antibiotic concentrations for FQM tested against the significant bacteriuria samples showed accurate AST results when compared with SDD. *Candida*, which can be associated with urine samples and which grows in various bacteriological media, did not quench fluorescein, apparently because of lack of production of 13-docosenamide or its analogues. Hence, the method appears to be specific to bacteria. When we tested FQM with different clinical isolates, a few *Pseudomonas* strains a non-fermenter of glucose, isolated from pus, blood-borne and CSF infections did not show the fluorescence quenching. However, some of these isolates showed fluorescence quenching after sub-culturing in Luria-Bertani medium The inability of *Pseudomonas* to catabolize glucose could have interfered in the production of the quencher compound. In such cases, the antibiogram can be determined by measuring the turbidity of the medium.

A major advantage of our method is the simplified instrumentation for quantitative optical imaging of fluorescence. Under the conditions standardized for the instrumentation, a CMOS-based camera could quantitatively record the intensity reduction of fluorescent green of the medium after the end of 8 h incubation. This is evident from the 100% correlation between the imager values and the commercial fluorescence reader values. Since the antibiogram device has the potential for several samples against many antibiotics (anywhere between 8–25 antibiotics), imaging platform for such operations need flexibility for scale-up. This design allows customization of antibiotic template to the need in terms of the number of antibiotics, number of samples and the types of antibiotics.

Though this new AST method has been tested on urine samples, a sterile biological fluid, the method can be readily adapted for positive blood cultures, cerebrospinal fluid, and other exudates. A full-fledged validation study is needed to expand such a scope. In case of a single type of bacterium present in the sample, the pattern is its antibiogram (as in this study). In case of more than one and predominantly two, the result is an aid for the right antibiotic therapy. FQM is a rapid AST compared to SDD and the testing time can be decreased (below 4 h) by mapping the fluorescence quenching pattern with hourly measurements. After completing AST by this method, identification of the pathogen, by the preferred biochemical methods can be performed with the cultured TSB-GF medium that has the high inoculum size after 6–8 h of growth.

Though the study had employed ATCC strains and clinical isolates with known identities for the sake of proving the feasibility of using in a clinical scenario, the actual use requires identification of the pathogen for deciding its breakpoints. Quicker pathogen identification methods in sterile biological fluids urine and serum are available now to identify syndrome-specific pathogens. Hence both pathogen identification and antibiogram results can be obtained within 8 h or 1 working day.

Quick spread of MDRs even among communities, especially through infections like UTIs, generation of contaminated clinical and hospital waste is an alarming concern not addressed adequately. FQM will reduce the waste by 10-folds for a typical test by using only 2.5 mL of liquid culture compared to 25 mL of contaminated agar from SDD. Based on the bill-of-material estimate of retail purchase, it is expected that FQM will drastically reduce the present cost of $5–10 per sample to <$2.50 per sample depending upon the number of antibiotics to be tested.

## Materials and Methods

### Antibiotics

The antibiotics for preparation of test plates were procured from the suppliers shown in parentheses: Amikacin, Ampicillin, Cefotaxime, Ceftriaxone, Ciprofloxacin, Gentamicin, Nitrofurantoin, Norfloxacin, Piperacillin/Tazobactam, Ofloxacin, (Sigma-Aldrich, USA), Amoxycillin/clavulanate (GlaxoSmithKline; Thane, India), Ceftazidime (VHB; Mumbai, India), Cefepime (Biocon; Bengaluru, India), Cefixime (GlaxoSmithKline; Thane, India), Colistin (Cipla; Pune, India), Erythromycin (Himedia; Mumbai, India), Imipenem (MSD; Mumbai, India), Levofloxacin (Alkem; Chennai, India), Meropenem (Cipla; Pune, India), Tetracycline (GlaxoSmithKline; Thane, India), Tigecycline (Natco; Chennai, India), Vancomycin (Himedia; Mumbai, India).

### Strains

American Type Culture Collection (ATCC) *Escherichia coli*: 25922, *Enterococcus faecalis:* 29212, *Klebsiella pneumoniae:* 700603, *Pseudomonas aeruginosa:* 27853, *Staphylococcus aureus:* 29213 were purchased from Microbiologics^®^ and maintained according to the supplier’s protocol.

### Growth media

Tryptic Soy Broth containing Glucose and Fluorescein (TSB-GF) was prepared by dissolving 17 grams of Tryptone, 3 grams of Soya bean meal, 5 grams of Sodium chloride, 2.5 grams of Dipotassium phosphate, 9 grams of Glucose in 1 L of distilled water and adding 25 µL of 100 mM fluorescein solution. Mueller Hinton (MH) broth No.2 Control Cations and Mueller Hinton Agar were purchased from Himedia and prepared according to the supplier’s protocol. All the preparations were autoclaved at 121 °C at 15 psi for 20 min.

### Evaluation of the fluorescence quenching method (FQM) with microdilution method using CLSI breakpoints

The antibiotic panel was prepared based on the CLSI quality control (QC) ranges for 10 antibiotics against reference ATCC cultures: *E. coli, E. faecalis, P. aeruginosa* and *S. aureus* (Table 1). Primary inoculum for each was prepared by suspending colonies of a single isolate, selected from an 18–24 h Muller Hilton agar plate culture, in MH broth. The suspension was adjusted to achieve turbidity equivalent to a 0.5 McFarland standard. This results in a suspension containing approximately 1–2 × 10^8^ colony forming units (CFU)/mL. Working stock of 5 × 10^5^ CFU/mL for each bacterium was prepared by diluting the primary inoculum in Mueller-Hilton medium for the CLSI broth microdilution method and TSB-GF for FQM respectively. Aliquots of 200 µL were dispensed into each of the antibiotic-coated wells of the microtitre plates and growth control wells. In the case of CLSI broth microdilution, OD_600 nm_ was taken after 24 h static incubation at 37 °C. For FQM, after 8 h of static incubation at 37 ^o^C, OD_600 nm_ and fluorescence were measured using Synergy H1 Hybrid Multi-Mode Reader (commercial reader). MICs determined from the results of both the methods for ATCC organisms tested against individual antibiotic was compared with the CLSI (2017) QC range. Consecutive trials were performed for 15 days for checking the consistency of the new test.

### FQM imager device

**Figure 2:**
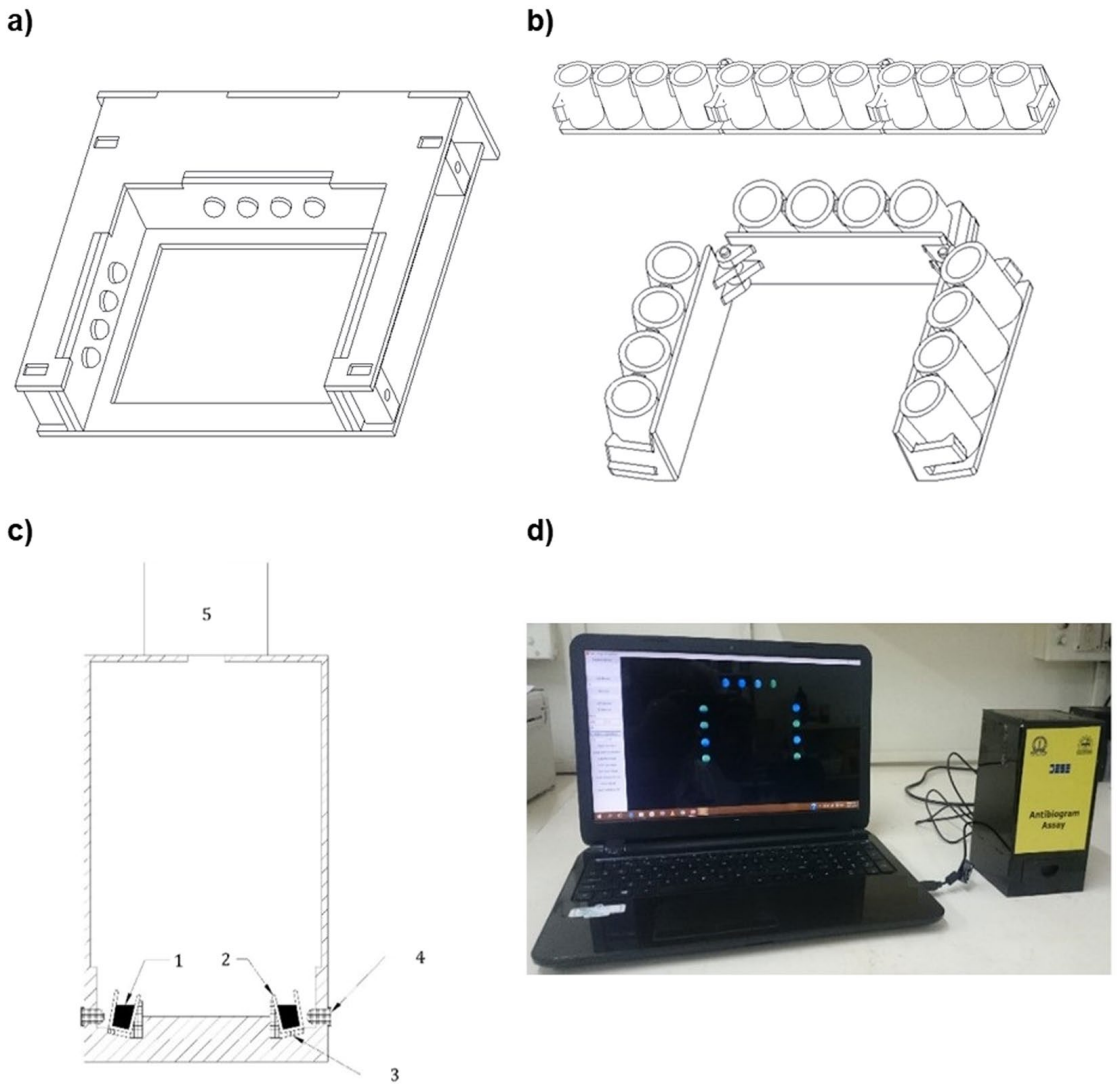
Schematic drawings of the design and the photo of optoelectronic imager device. (**a**) Illumination setup with LEDs for excitation of fluorescein (**b**) The 12-well microtitre strip and its folded configuration within the strip holder (**c**) Vertical cross-sectional diagram of the imager setup (1) FQM strips with sample (2) and (3) Strip holder loaded into the Imager (4) LEDs exciting the fluorescein in the medium (5) Camera position (**d**) Antibiogram Imager device connected to a computing device to capture and process the image of fluorescence of TSB-GF medium within the wells.

The in-house fabricated optoelectronic device housed in a black box measuring 10 cm in length 9.6 cm breadth and 20 cm in height consists of an illumination (Fig. 2a) and sample loading setup at the bottom and the CMOS-based image acquisition setup on the top (Fig. 2c(5)). The illumination setup consists of a single blue LED positioned for each well of the 12-well microtitre strip (Fig. 2c(4)) excites the medium fluorescein in the well. A strip-loading tray with a sliding mechanism at the base (Fig. 2c(3)) aligns the samples with respective blue LEDs. The camera captures the image of the strip and transfers the data to a computing device via a USB port (Fig. 2d). An in-house developed software installed in the computing device connected to the imager controls the image capture and processing operation. The software is based on an algorithm that quantifies the amount of green colour in the medium. As fluorescent medium (uncultured sample or sample sensitive to antibiotic) and the quenched medium (cultured sample or sample resistant to antibiotic) formed well-separated clusters in image processing quantification, the algorithm is coded to identify growth as antibiotic resistance and no growth as antibiotic susceptibility. Based on which the AST report is generated and securely transferred to the clinical lab/hospital/doctor.

### Preparation of antibiotic coated microtitre wells

Detachable 12-well microtiter strips, which are commercially available as 8 strips (96-wells) mounted on a holding frame, were used to prepare the antibiotic coated plate for testing the clinical samples. A panel of 21 antibiotics at CLSI breakpoint concentrations for susceptible, intermediate and resistance were used. A fixed aliquot (10 µL) of the respective antibiotic working stocks prepared with sterile pyrogen-free water were delivered into A1-F3 wells of the strips according to the final concentration as shown in the template (Table 4). The prepared antibiotic strips were freeze-dried at −110 °C and stored at −80 °C until use.

**Table 4:** Template of the antibiogram with an indication of the final antibiotic concentration (values in mg/L as the final concentration in tests) coated to the wells of microtitre strips for FQM kit used in the clinical validation study for comparing with SDD method.

	1	2	3	4	5	6	7	8	9	10	11	12
A	AMI 8	AMI 16	AMI 64	AMC 8	AMC 16	AMC 32	AMP 4	AMP 8	AMP 32	CEF 0.12	CEF 0.25	CEF 1
B	FEP 2	FEP 8	FEP 32	TAZ 2	TAZ 8	TAZ 32	AXO 2	AXO 16	AXO 64	CIP 0.5	CIP 2	CIP 4
C	COL 0.25	COL 1	COL 4	ERY 0.25	ERY 1	ERY 4	GEN 2	GEN 4	GEN 16	HGN 4	HGN 8	HGN 32
D	IMI 2	IMI 4	IMI 16	LEVO 1	LEVO 3	LEVO 8	MERO 2	MERO 4	MERO 16	NIT 16	NIT 32	NIT 128
E	NOR 1	NOR 3	NOR 8	P/T4 16/4	P/T4 32/4	P/T4 128/4	TET 2	TET 4	TET 16	TGC 1	TGC 3	TGC 8
F	VAN 0.25	VAN 3	VAN 32	Medium Ctrl	Medium Ctrl	Growth Ctrl	Growth Ctrl					
G												
H												

### Performing Fluorescence Quenching Method (FQM) with urine samples

For the FQM method, 150 µL of patient urine sample was inoculated in 15 mL TSB-GF medium. 200 µL aliquots of the inoculated medium was dispensed into A1-F3 wells (antibiotic-coated microwells) & F6, F7 (no antibiotic)-growth control wells and 200 µL of sterile medium were dispensed in F4, F5-medium control wells of the strips. The strips were covered with transparent adhesive tape to prevent spillage or contamination. After 8 h incubation, each 12-well strip of the test plate was snapped sequentially into three 4-well pieces (after cutting the adhesive tape between the segments to enable folding) and placed in the receptacle as shown in Fig. 2b (top). It was then folded into inverted U shape (Fig. 2b, bottom), placed in the tray and inserted into the device so as to align with the illumination unit (Fig. 2a–c) of the camera connected to a computing device (Fig. 2d). The software installed on the computing device captured and processed the image of the strips into susceptibility results based on the green intensity of the wells. The susceptibility report can be communicated to the clinical lab/hospital/doctor directly.

### Comparison of FQM results with those of Standard Disk Diffusion (SDD) method using urine samples

The patient urine sample received for routine culture-and-susceptibility testing were selected for antibiotic susceptibility testing using SDD and FQM. The urine samples were analyzed using UROQUATTRO, HB&L™ and sterile urine sample, urine sample infected with *Candida* spp. and significant bacteriuria samples infected with only a single type of organism were preferred. For SDD method, after the analysis, the bacterium was isolated from the urine sample by plating on MH agar and identified by Gram staining. The isolates were then used as inoculum and hence, AST was performed 24 h after sample collection. For FQM, the corresponding urine immediately after UROQUATTRO, HB&L™ analysis was used as inoculum. For SDD method, the zone of inhibition was measured between 18–24 hours of aerobic incubation at 37 °C. The result of FQM method was generated using the fluorescence imager device after 8 h aerobic incubation at 37 °C. The antibiotic susceptibility results of both the methods were compared and the results were classified according to Table 5.

**Table 5:** The categories for the comparative antibiotic susceptibility results of SDD and FQM method are classified under.

**Match**	Absolute correlation between the susceptibility pattern of FQM method and SDD method (Sensitive, Intermediate and Resistant)
**Minor Error**	Discrepancy between FQM method and SDD method by one interpretive category (Sensitive or Resistant by SDD method whereas Intermediate by FQM method; Intermediate by SDD method whereas Sensitive or Resistant by FQM method)
**Major Error**	Result was reported as Sensitive by SDD method and Resistant by FQM method
**Very Major Error**	Result was reported as Resistant by SDD method and Sensitive by FQM method

### Ethical Declarations

The samples used in this study are leftover non-identified clinical urine samples thus this study is categorized under “Less than minimal risk”. Hence, this study is exempted from ethical review according to “National Ethical Guidelines for Biomedical and Health Research Involving Human Participants – Indian Council of Medical Research”. The authors Rohit Radhakrishnan, J. Rajesh, Dr N. S. Dinesh, Dr C. P. Thangavelu, and Dr K. Sankaran declare that the FQM-AST results of this study did not influence the antibiotic therapy provided to the patients.
